# Hidden Information Revealed Using the Orthogonal System of Nucleic Acids

**DOI:** 10.3390/ijms23031804

**Published:** 2022-02-04

**Authors:** Viktor Víglaský

**Affiliations:** Department of Biochemistry, Institute of Chemistry, Faculty of Sciences, Pavol Jozef Šafárik University, 04001 Košice, Slovakia; viktor.viglasky@upjs.sk; Tel.: +421-55-2341262

**Keywords:** orthogonal representation, G-quadruplex, G4Hunter, algorithm

## Abstract

In this study, the organization of genetic information in nucleic acids is defined using a novel orthogonal representation. Clearly defined base pairing in DNA allows the linear base chain and sequence to be mathematically transformed into an orthogonal representation where the G–C and A–T pairs are displayed in different planes that are perpendicular to each other. This form of base allocation enables the evaluation of any nucleic acid and predicts the likelihood of a particular region to form non-canonical motifs. The G4Hunter algorithm is currently a popular method of identifying G-quadruplex forming sequences in nucleic acids, and offers promising scores despite its lack of a substantial rational basis. The orthogonal representation described here is an effort to address this incongruity. In addition, the orthogonal display facilitates the search for other sequences that are capable of adopting non-canonical motifs, such as direct and palindromic repeats. The technique can also be used for various RNAs, including any aptamers. This powerful tool based on an orthogonal system offers considerable potential for a wide range of applications.

## 1. Introduction

As is well known, DNA molecules often occur in an antiparallel double-stranded structure due to Watson−Crick (WC) base pairing, with adenine and guanine bases pairing with thymine and cytosine, respectively. A unique feature of these molecules is their ability to pair not only through WC pairing, but also through Hoogsteen bonds. Non-canonical structures can be stabilized by a combination of both types of hydrogen bonds and can also contain several unpaired bases, such as G-quadruplex, i-motif, triplexes, hairpin, and cruciform [1,2]. Non-canonical structures exist in cells and play important roles in gene expression regulation [1].

**Figure 1:**
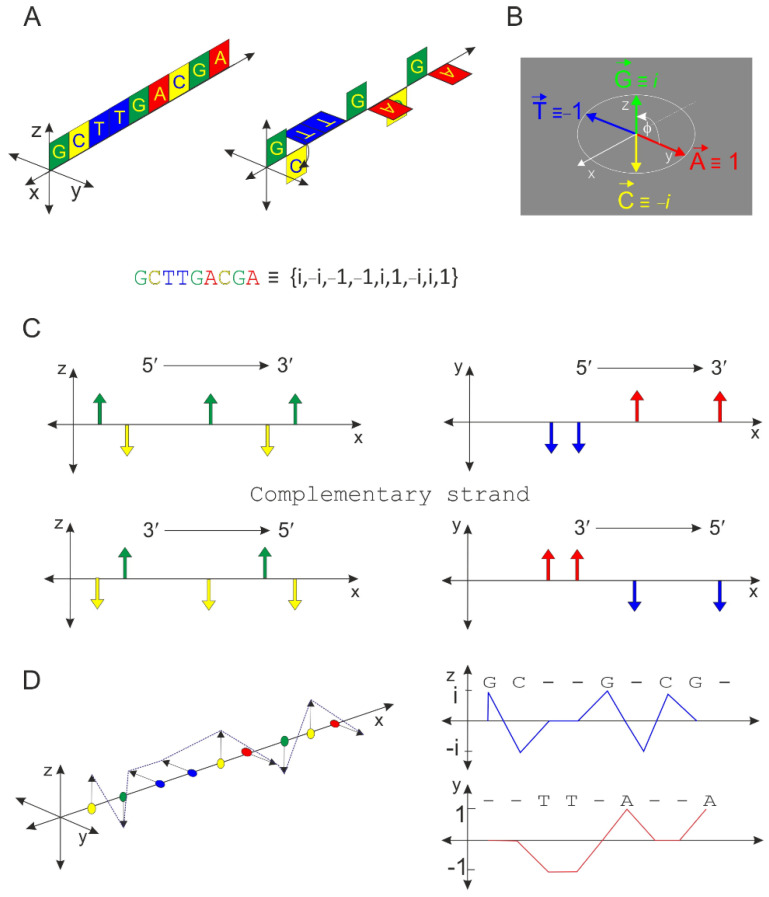
Basic properties of an orthogonal system. Standard sequence visualization is performed on two perpendicular planes, where nucleotides A + T are on the xy planes and C + G are on the xz planes. The nucleotide order is expressed by an integer value on the *x*-axis (**A**). There is a close analogy with the representation of complex integers (**B**), and a unit circle is used for this purpose. In the complex space, any oligonucleotide in the DNA sequence can be expressed instead of A, T, C, and G by four values: −1, 1, −i, and i, respectively. The complementary strand of DNA is a mirror image of the original sequence on a given plane of display (**C**). The sequence can be displayed in a complex space using vectors that can be projected into a real or complex plane (**D**).

Nucleic acid consists of building blocks of nucleotides that are arranged in different permutations, with the order of the nucleotides determining the sequence of DNA or RNA molecules (Figure 1A). The nucleic acid sequence is crucial for the arrangement of amino acids in proteins and the 3D structure of RNA, which does not necessarily translate into protein. These sequences are not coincidental. DNA consists of characteristic sequence motifs typical of any organism, usually untranslated, that play key roles at various levels of gene expression [3,4,5]. For example, they separate coding and non-coding regions, control the efficiency of promoter sequences, segment chromosomes, and signal for transcription and translation machineries. Countless other examples are known where specific sequence motifs play a key role in regulating the gene expression and the cell signaling system [2]. 

The identification of sequence clusters and their mutations is particularly useful for understanding the expression of structural genes, which are responsible for various pathological manifestations. An awareness of the DNA sequence alone is not sufficient to provide a full understanding of these processes, and therefore a number of diverse bioinformatic approaches have been developed that enable the identification of so-called non-standard sequences in the genome. The dramatic increase in the accumulation of genomic data over the last decade poses a considerable challenge in terms of processing and provides an opportunity to develop computational analyzes that are capable of sophisticated screening processes of unknown genomes, including their graphical representation [6]. 

The approach known as “digital signal processing” has seen increasing use in genomic DNA research as a means of revealing genome structures and identifying hidden periodicities and features that cannot be determined using conventional DNA symbolic and graphical representation techniques [6]. Various numerical, vector, color, and different graphical representation of nucleobases in DNA have already been described in earlier studies [6,7,8,9,10,11,12]. For canonical putative sequences adopting cruciform or G-quadruplex structures, it is more appropriate to use an application specially tailored for this purpose, for example, computational approaches, which study these motifs to allow for a detailed analysis of the genomes [13,14,15,16,17].

Interestingly, the G4Hunter algorithm offers one of the highest search scores for identifying sequences that form G-quadruplexes, but there is still a lack of a rational explanation for this success rate. The G4Hunter algorithm considers the G-richness and G-skewness of a given sequence, and provides a quadruplex propensity score as an output. The searching strategy is simple; each position in a sequence is given a score between −4 and 4. Scores of 0 indicate A and T, while positive scores indicate G and negative scores C. A single G achieves a score of 1, and two, three, and four neighboring Gs scores of 2, 3, and 4, respectively; a score of 4 also suggests the presence of higher numbers of Gs. The C bases are scored similarly, but all of the values are negative [18]. The G4Hunter algorithm also retains some G–C pairing features; the G score has the opposite value of C, but not in the case of A–T pairing. This study will present an alternative to the G4Hunter approach. In this system, the basic attribute related to base pairing is preserved for both WC base-pairs. Although the basic principle of the system is very simple, it does not appear to have been described before.

## 2. Results and Discussion

### 2.1. Principle of the Orthogonal Algorithm

The principle of the algorithm is shown in Figure 1. Complementary oligonucleotides are shown in the following colors: A—red; T—blue; G—green; C—yellow. A + T bases occur only on the xy planes and C + G bases only on the xz planes. The representative sequence is d(GCTTGACGA) (panel A). There is a close analogy with the representation of complex numbers, and it is therefore possible to state that A + T are projected in the real plane and G + C in the imaginary plane (panel B). Based on this analogy, the values 1, −1, i, and −i can be assigned to the individual nucleotides A, T, G, and C, respectively. If the size of vectors A, T, G, and C are equivalent and equal to 1, then the endpoint of each vector lies on the unit circle, and it is possible to express a representative sequence using a linear string {i, −i, −1, −1, i, 1, −i, i, 1}. Any DNA sequence can be divided into real and imaginary components, but both categories are coupled. The definition of the axes is variable but due to the symmetry of this view, similar results would also be obtained with a different choice of planes and axes. In principle, only a single condition is required to be met; C must be opposite G, and A must be opposite T. An antiparallel strand represents a mirror image for both components (panel C). The vector representation and projection into real and imaginary planes are shown in panel D. In situations when it cannot be ruled out that the individual endpoints of vectors A, T, C, and G lie on an ellipse and that the angle φ is not exactly 90 degrees, the quantitative results will offer an even more reliable score than a purely orthogonal system for sequences forming a specific non-canonical motif (see below).

**Figure 2:**
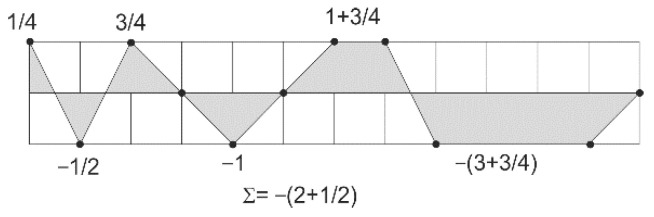
Calculation of the area in one of the planes determined by the projection of a specific sequence.

The profile of projection into the plane is given by the sequence, and an example of this is shown in Figure 2. The projection shows the following two sets of sequences: ATA(G/C)T(G/C)AATTTT(G/C) and GCG(A/T)C(A/T)GGCCCC(A/T). The area is not solely dependent on a given nucleotide, but is also influenced to some extent by the neighboring nucleotides. For example, the area in the xy plane given by the C**A**C sequence is equal to 1, the T**A**T is equal to 0.5, and the C**A**T is 0.75. The total area of a given sequence in Figure 2 in one of the projection planes, which achieves a negative value of −2.5. An important parameter is obtained if this value is divided by the number of nucleotides. 

### 2.2. G-Quadruplex Forming Sequences and Non-Canonical Motifs

**Figure 3:**
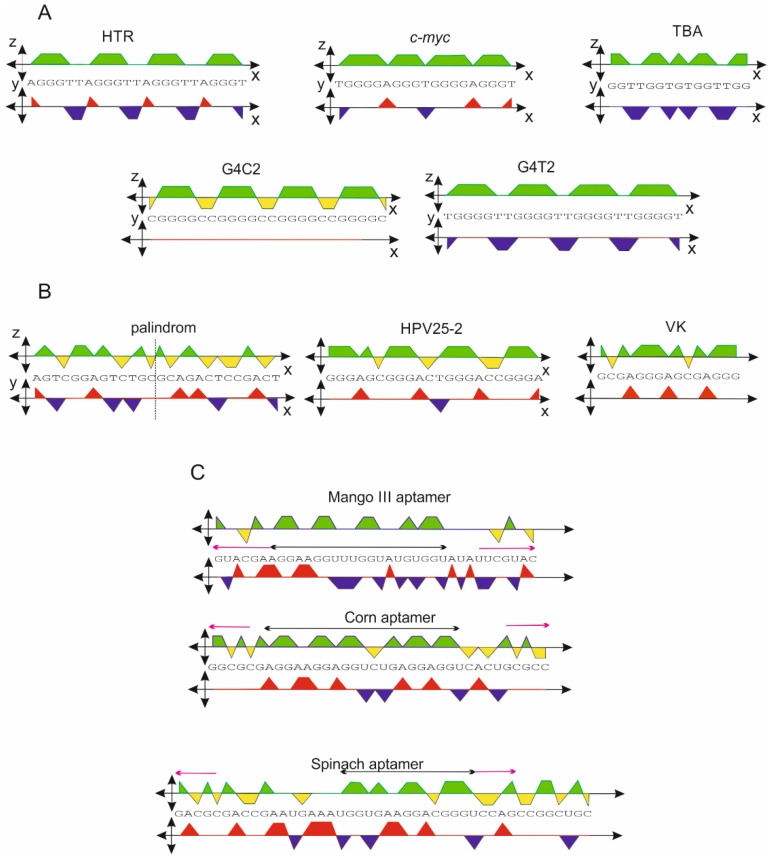
(**A**) Orthogonal projection of sequences adopting G-quadruplexes: human telomeric repeats (HTR), *c-myc* promoter sequence, and thrombin binding aptamer (TBA). (**B**) Example of a palindromic sequence adopting a hairpin and sequences adopting other non-canonical structures. (**C**) Sequence of RNA aptamers Mango III and Corn adopting a structure consisting of both G-quadruplex and dsRNA.

The orthogonal system was applied to a series of sequences that are known to be capable of forming a G-quadruplex motif. Five examples of G-quadruplex sequences, human telomeric repeats (HTR), c-myc promoter sequence, thrombin binding aptamer (TBA), d[(C(G_4_C_2_)_3_G_4_C], and d[T(G_4_T_2_)_3_G_4_T] are shown in Figure 3A. Each of the DNA sequences is capable of forming a relatively stable G-quadruplex structure in the presence of a potassium ion [19,20,21,22,23,24,25]. This set of sequences is displayed in the xz-projection. 

The areas of green projection for HTR, *c-myc*, and TBA are 12, 14, and 8, respectively. The orthogonal system provides the following scores: 0.52, 0.74, and 0.53, respectively. In contrast, the G4Hunter scores are as follows: 1.57, 2.11, and 2.2, respectively. However, if the radius “r” of the circle is equal to 3, as shown in Figure 1, then the scores multiplied by a factor of 3 provide values of 1.56, 2.22, and 1.59, respectively, with the first two values being very close to those obtained using the G4Hunter algorithm (Table 1). The scores for G4C2 and G4T2 give values of 1.13 and 2.0, while those obtained from G4Hunter are 2.08 and 2.67. The deviation between these types of algorithms is a result of the overly strong parameterization in G4Hunter in cases of two, four, or more adjacent Gs. The orthogonal projection and G4Hunter algorithm provide similar results for sequences consisting of less than four contiguous Gs.

The HPV25-2 and VK (pdb ID: 2MJJ) sequences are known not to form G-quadruplexes [26,27], and the scores for these sequences are 1.16 and 1.10 for HPV25-2 and VK, respectively. However, the G4hunter algorithm gives a false positive score, indicating that the sequences have the capacity to form a G-quadruplex structure. If the score obtained by the orthogonal system falls within the range of 1.1–1.2, the prediction of G-quadruplex formation can be somewhat ambiguous.

However, if the score obtained from the xy projection does not show higher positive values, then the sequence still has the potential to adopt a G-quadruplex structure, but experimental verification would be recommended to confirm the formation of a G-quadruplex from the sequence in such a case. In essence, an increasing number of As in a sequence reduces the inclination to adopt G-quadruplex, mainly if the xz-score is less than 1.2. Therefore, the G4C2 sequence listed in Table 1 does not lose the potential to form a G-quadruplex, even at a lower xz-score of 1.13, but this is not the case for the VK and HPV25-2 sequences. For example, while the d(G_3_A_2_)G_3_ sequence still has the potential to form a G-quadruplex with xz- and xy-scores of 1.83 and 1.00, respectively, the CD spectrum results (not shown in this study) do not confirm the formation of the G-quadruplex structure of the sequence d(G_3_A_3_)G_3_ with xz- and xy scores of 1.57 and 1.29, respectively, These findings would suggest that the xz-score alone may not be a sufficient indicator to confirm the actual presence of G-quadruplexes. 

Even more interesting results were obtained in the case of the two RNA aptamers Mango III and Corn [28,29]. The orthogonal system is not only applicable for DNA sequences, but it can also be expanded for use with RNA molecules, with the U being used instead of T with the same value. The central sequence scores (cs) obtained for these aptamers are highlighted by black double-arrows in Figure 3C, and the values are shown in Table 1. The G4Hunter algorithm failed for both aptamers, with no G-quadruplex formation predicted, but the orthogonal system did predict G-quadruplex formation, with a xz-score higher than 1.2. In addition, clear palindromic regions were identified, highlighted with the purple arrows in Figure 3. Such a complex view of a given sequence clearly suggests that a G-quadruplex could form in the central region and that the terminal sequences would also be paired. The 3D structures of these aptamers only confirm these predicted results (pdb ID: 6E80 and 6E8T). 

**Figure 4:**
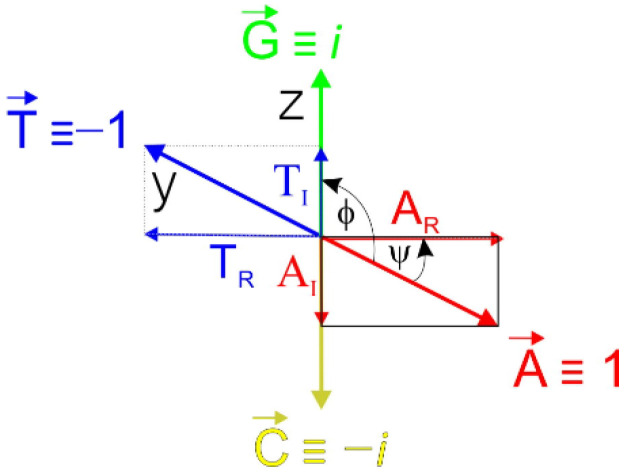
A quasi-orthogonal system in which the xy plane rotates around the x-axis at angle ψ. The projection of the vector A and T into imaginary and real components is also shown. Imaginary components contribute in the xz planes to the C + G score.

We accept that the orthogonal system is not a completely perfect method, but the accuracy can be increased if the orthogonality is slightly disturbed, resulting in a reduction in the number of false positives. The generalization of the system shown in Figure 1B is such that no nucleotide needs to be defined as a purely real or imaginary number; their coordinates lie on a circle or ellipse, depending on constants r_1_ and r_2_. For the sake of simplicity, these constants were equal to 1. If the condition of complementarity is maintained, the coordinates [y, z] for A, T, C, and G vectors can generally be expressed as follows:

A = r_1_.[cos (α); isin(α)],

T = r_1_.[cos (α + π); isin(α + π)],

G = r_2_.[cos (β); isin(β)],

C = r_2_.[cos(β + π); isin(β + π)],

where r_1_ and r_2_ are variable constants (radius), and the difference α–β expresses the angle *φ* between vectors A and G or C and T. If the angular difference is greater than 90° than angle ψ, then the contribution of the imaginary components for A directly reduces the score in the imaginary plane (xz)*,* Figure 4. 

The result is a decrease in the probability of G-quadruplex formation. As has been shown previously, the HPV25-2 and VK sequences show a significant signal from A-nucleotides [26,27]. On the other hand, the presence of Ts increases the probability of G-quadruplex formation. The value of angle ψ can be estimated from the experimentally confirmed sequences forming a G-quadruplex in which the orthogonal scores are ambiguous. The scores recalculated for two different values of angle ψ, 15° and 30°, are also shown in Table 1. Implementing this correction results in a significant reduction in ambiguity. The ψ around 30° seems to be more ideal, with the threshold for G-quadruplex formation approaching 1.1. This so-called ψ-correction has been applied to more than 100 experimentally validated sequences that have adopted the G-quadruplex structure, but no exception has been found to date.

### 2.3. Genetic Code in Orthogonal Presentation

The system presented here can be applied to all sizes of nucleic acids, including short oligonucleotides. Recent research has revealed that short sequence regions often play a key role; for example, they are a target for many proteins and they are recognized by various restriction enzymes, transcription factors, and ribosomes. It is clear that short trinucleotide sequences are sufficient to encode amino acids in the form of a genetic code. The numerical transformation of the genetic code into an orthogonal system is shown in Table 2.

**Figure 5:**
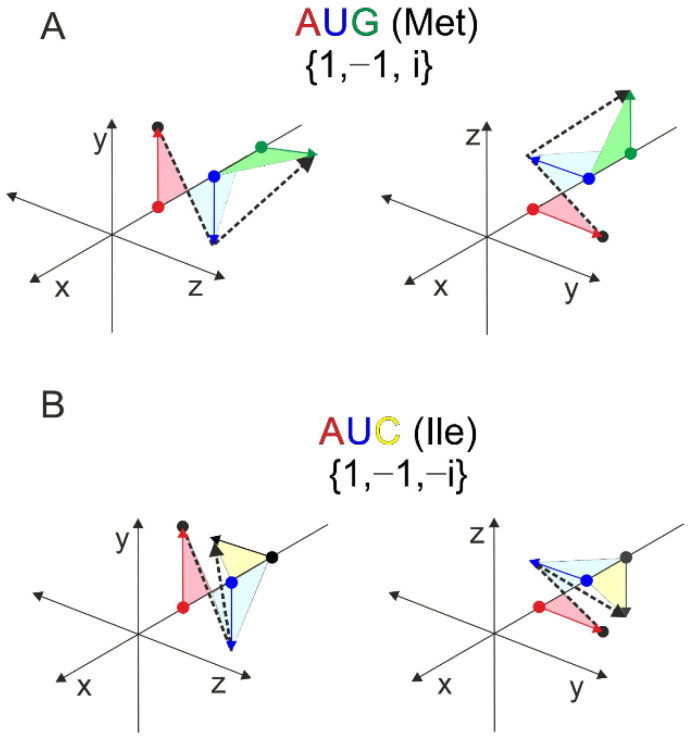
Orthogonal projection of two codons: Met (**A**) and Ile (**B**). The difference is found in the third base. The corresponding polylines are mirrored in the orthogonal projection (dashed lines).

The 3D examples of the two mirror codons, the start codon-methionine and isoleucine are shown in Figure 5. Each pair of graphical representations is equivalent, the only difference being that they are shown from a different angle. Any triplet-nucleotide sequence can be represented by a single line (dashed lines). These types of graphical and numerical representations could be of considerable use in bioinformatic analyses [30].

**Figure 6:**
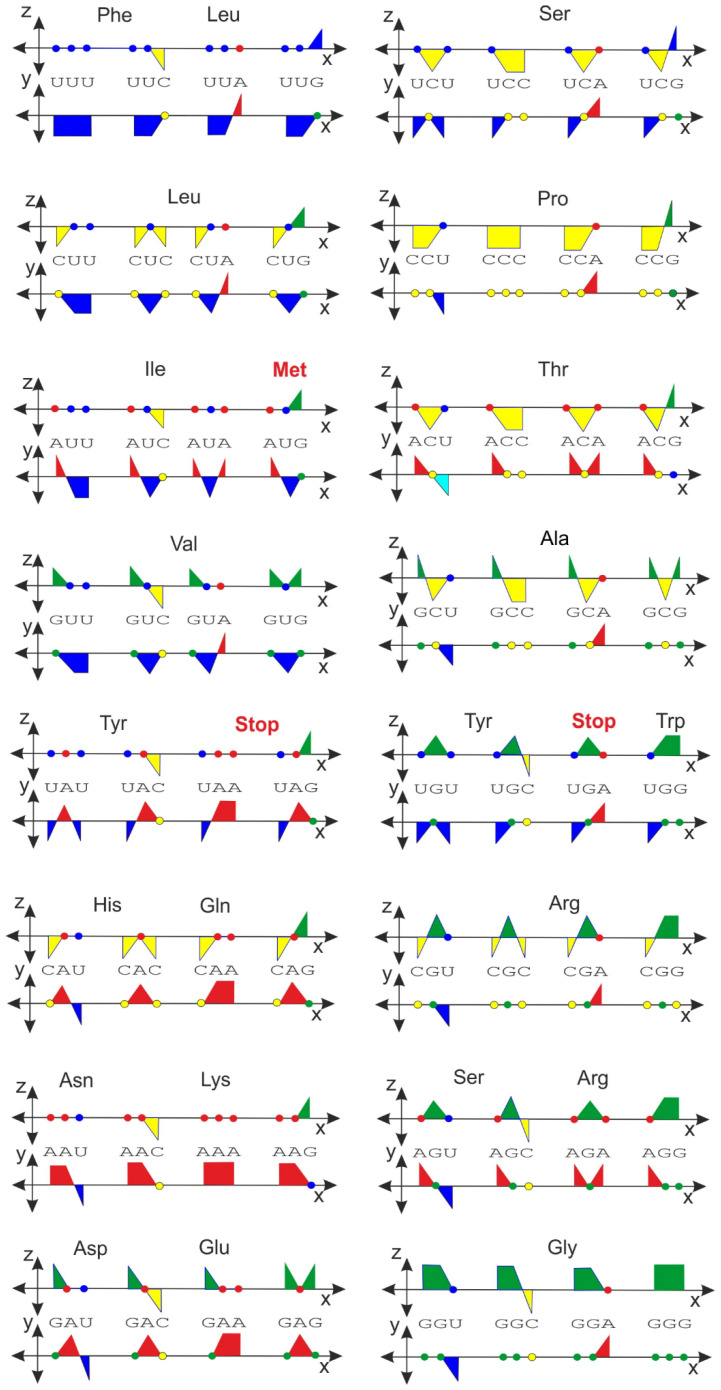
Genetic code represented in the orthogonal system. Each codon is projected into both the xy- and xz-planes.

An even more interesting representation, analogous to the previous application for the DNA and RNA sequences, is shown in Figure 6. There is no ambiguity concerning which color is dominant for a particular group of codons. The different color coding of the codon tetrahedral representation has also been performed and described in a previous study, although the strategy used in that case was based on a slightly different but still complex basis [7]. Nevertheless, the orthogonal representation method is a simpler technique and can also be transformed into a tetrahedral representation.

**Figure 7:**
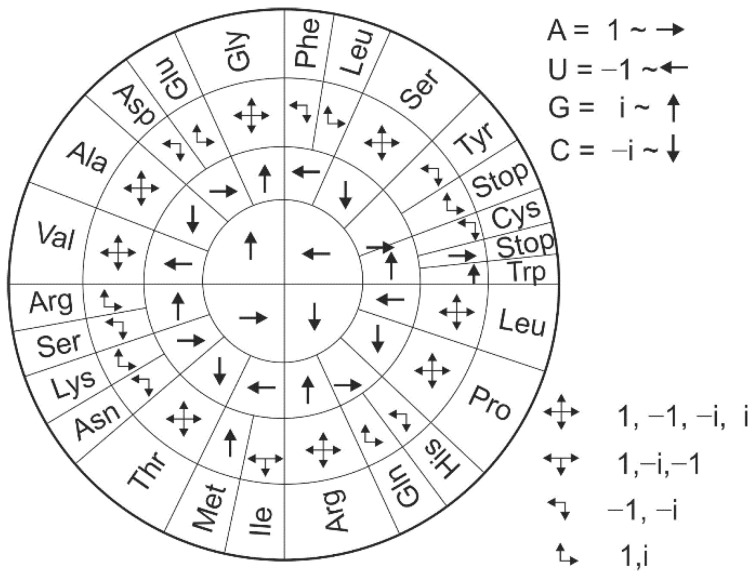
Vector representation of a genetic code.

The vector representation derived from the orthogonal system offers an alternative view on the genetic code (Figure 7). Interestingly, some combinations of double degeneracy in the third codon base for a single amino acid, specifically a combination of CG (−i, i) or UA (−1, 1), are not permitted. No amino acid is specified by these combinations, except those that are more degenerate than Gly, Ser, Leu, Pro, Arg, Ile, Thr, Val, and Ala.

**Figure 8:**
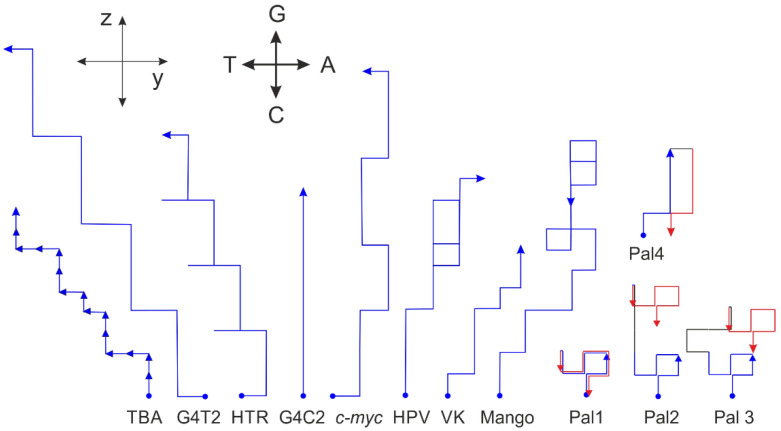
Selected sequences represented by orthogonal vector analysis. The direction of each oligonucleotide is determined by the vector, analogous to that defined in Figure 7. Each oligonucleotide is represented by arrows, analogical to that used in Figure 7. Traces corresponding to sequences forming G-quadruplexes do not tend to point more significantly to the right. The first half trajectory (blue) of the palindromic sequence (Pal) is identical to that of the second (red). Spacers are shown in black lines.

Analogically, the vector representation is also applicable for longer sequences. The sequences used for the projection in the xy- and xz-planes shown in Figure 3 are displayed in the vector representation in Figure 8. Again, the fact that G-quadruplexes show some features is confirmed. The sequences adopting biologically relevant G-quadruplexes also show a tendency not to turn right, a feature that may suggest that many As can exert some destabilization effect on G-quadruplex formation. If the start and end points in this presentation of the trajectory are identical, then the sequence consists of the same number of As and Ts and the same number of Gs and Cs. If the second half of the trajectory is identical to the first, then the sequence is a perfect palindrome, e.g., Pal1: d (GAGTCTGCAGACTC). However, the start and end points of imperfect palindromic sequences are not identical. Irrespective of the central sequence, which is not part of the palindromic region (black lines), the trajectory consists of two antiparallel sections, e.g., Pal2: d(GAGTCTGgggCAGACTC), Pal3: d(GAGTCTGtgaagCAGACTC) and Pal4: d(GAGGGaCCCTC).

## 3. Concluding Remarks

The orthogonal system can easily be used for all types and sizes of nucleic acids. It can be adapted to search for tandem forward and inverse repeats, and is, of course, ideal for sequences featuring non-canonical motifs. An indirect side effect of the method is that this presentation offers a rational explanation of why the G4Hunter algorithm provides such promising scores for i-motifs and G-quadruplexes. In addition, the system also explains the weaknesses of the G4Hunter algorithm. An orthogonal system allows any nucleic acid sequences to be presented in numerical, color, and vector representations. The system is particularly efficient at identifying sequential domains responsible for a wide range of biological functions. Nevertheless, a deviation from orthogonality offers a significant improvement in the prediction of G-quadruplex adoption from a specific sequence. Although the quasi-orthogonal system loses its perfect symmetry, it allows for the possibility of distinguishing between G-quadruplexes consisting of loops featuring pure As or Ts nucleotides, a feature which is not possible with the G4hunter algorithm and the orthogonal system. For example, this system would explain why the presence of As reduces the likelihood of G-quadruplex formation.

## Figures and Tables

**Table 1:** Scores obtained with the orthogonal presentation and G4Hunter algorithm.

Sequence	xz-Projection Area, r = 3	xy-Projection Area, r = 3	G4hunter Score	xz-Projection Area, r = 3, ψ = 15° ^#^	xz-Projection Area, r = 3, ψ = 30° ^#^	G4 Formation
HTR	1.56	−0.39	1.57	1.66	1.75	yes
*c-myc*	2.22	0	2.11	2.22	2.22	yes
TBA	1.59	−1.2	2.20	1.90	2.19	yes
G4C2	1.13	0	2.08	1.13	1.13	yes
G4T2	2.00	−0.87	2.67	2.23	2.43	yes
HPV25-2	1.16	+0.34	1.68	1.07	0.99	no
VK	1.1	+0.60	1.4	0.94	0.80	no
palindrom *	0	0	0	0	0	no
cs-Mango III	1.35	−0.41	0.85	1.46	1.55	yes
cs-Corn	1.41	+0.18	0.94	1.36	1.32	yes

*—any sequences where the number of As and Ts is equal and the number of Gs and Cs then the score is 0; special cases include any perfect palindrom; see also Figure 8; ^#^—ψ-correction for quasi-orthogonal system.

**Table 2:** Genetic code in numeric representation, radius r equals 1.

**Ala/A**	GCU {i, −i, −1}	GCC {i, −i, −i}	GCA {i, −i, 1}	GCG {i, −i, i}		
**Arg/R**	CGU {−i, i, −1}	CGC {i, i, −i}	CGA {−i, i, 1}	CGG {−i, i, i}	AGA {1, i, 1}	AGG {1, i, i}
**Asn/N**	AAU {1, 1, −1}	AAC {1, 1, −i}				
**Asp/D**	GAU {i, 1, −1}	GAC {i, 1, −i}				
**Cys/C**	UGU {−1, i, −1}	UGC {−1, i, −i}				
**Gln/Q**	CAA {−i, 1, 1}	CAG {−i, 1, i}				
**Glu/E**	GAA {i, 1, 1}	GAG {i, 1, i}				
**Gly/G**	GGU {i, i, −1}	GGC {i, i, −i}	GGA {i, i, 1}	GGG {i, i, i}		
**His/H**	CAU {−i, 1, −1}	CAC {−i, 1, −i}				
**Ile/I**	AUU {1, −1, −1}	AUC {1, −1, -i}	AUA {1, −1, 1}			
**Leu/L**	UUA {−1, −1, 1}	UUG {−1, −1, i}	CUU {−i, −1, −1}	CUC {−i, −1, −i}	CUA {−i, −1, 1}	CUG {−i, −1, i}
**Lys/K**	AAA {1, 1, 1}	AAG {1, 1, i}				
**Met/M**	AUG {1, −1, i}					
**Phe/F**	UUU {−1, −1, −1}	UUC {−1, −1, −i}				
**Pro/P**	CCU {−i, −i, −1}	CCC {−i, −i, −i}	CCA {−i, −i, 1}	CCG {−i, −i, i}		
**Ser/S**	UCU {−1, −i, −1}	UCC {−1, −i, −i}	UCA {−1, −i, 1}	UCG {−1, −i, i}	AGU {1, i, −1}	AGC {1, i, −i}
**Thr/T**	ACU {1, −i, −1}	ACC {1, −i, −i}	ACA {1, −i, 1}	ACG {1, −i, i}		
**Trp/W**	UGG {−1, i, i}					
**Tyr/Y**	UAU {−1, 1, −1}	UAC {−1, 1, −i}				
**Val/V**	GUU {i, −1, −1}	GUC {i, −1, −i}	GUA {i, −1, 1}	GUG {i, −1, i}		
**STOP**	UAG {−1, 1, i}	UGA {−1, i, 1}	UAA {−1, 1, 1}			

## Data Availability

The data presented in this study are available in the article.

## References

[1] Takahashi S., Sugimoto N. (2020). Stability prediction of canonical and non-canonical structures of nucleic acids in various molecular environments and cells. Chem. Soc. Rev..

[2] Takahashi S., Sugimoto N. (2021). Roles of non-canonical structures of nucleic acids in cancer and neurodegenerative diseases. Nucleic Acids Res..

[3] Weiner A.M. (2002). SINEs and LINEs: The art of biting the hand that feeds you. Curr. Opin. Cell Biol..

[4] Siggers T., Gordân R. (2014). Protein-DNA binding: Complexities and multi-protein codes. Nucleic Acids Res..

[5] Balasubramaniyam T., Oh K.I., Jin H.S., Ahn H.B., Kim B.S., Lee J.H. (2021). Non-Canonical Helical Structure of Nucleic Acids Containing Base-Modified Nucleotides. Int. J. Mol. Sci..

[6] Roy A., Raychaudhury C., Nandy A. (1998). Novel techniques of graphical representation and analysis of DNA sequences—A review. J. Biosci..

[7] Cristea P.D. (2002). Conversion of nucleotides sequences into genomic signals. J. Cell. Mol. Med..

[8] Cristea P.D., Dougherty E.R. (2005). Representation and analysis of DNA sequences. Genomic Signal Processing and Statistics: EURASIP Book Series in Signal Processing and Communications.

[9] Mendizabal-Ruiz G., Román-Godínez I., Torres-Ramos S., Salido-Ruiz R.A., Morales J.A. (2017). On DNA numerical representations for genomic similarity computation. PLoS ONE.

[10] Voss R.F. (1992). Evolution of long-range fractal correlations and 1/f noise in DNA base sequences. Phys. Rev. Lett..

[11] Kwan H.K., Arniker S.B. Numerical representation of DNA sequences. Proceedings of the 2009 IEEE International Conference on Electro/Information Technology.

[12] Benson G. (1999). Tandem repeats finder: A program to analyze DNA sequences. Nucleic Acids Res..

[13] Brázda V., Kolomazník J., Lýsek J., Hároníková L., Coufal J., Št’astný J. (2016). Palindrome analyser—A new web-based server for predicting and evaluating inverted repeats in nucleotide sequences. Biochem. Biophys. Res. Commun..

[14] Brazda V., Kolomaznik J., Mergny J.L., Stastny J. (2020). G4Killer web application: A tool to design G-quadruplex mutations. Bioinformatics.

[15] Brázda V., Kolomazník J., Lýsek J., Bartas M., Fojta M., Šťastný J., Mergny J.L. (2019). G4Hunter web application: A web server for G-quadruplex prediction. Bioinformatics.

[16] Kikin O., D’Antonio L., Bagga P.S. (2006). QGRS Mapper: A web-based server for predicting G-quadruplexes in nucleotide sequences. Nucleic Acids Res..

[17] Oh K.I., Kim J., Park C.J., Lee J.H. (2020). Dynamics Studies of DNA with Non-canonical Structure Using NMR Spectroscopy. Int. J. Mol. Sci..

[18] Bedrat A., Lacroix L., Mergny J.L. (2016). Re-evaluation of G-quadruplex propensity with G4Hunter. Nucleic Acids Res..

[19] Macaya R.F., Schultze P., Smith F.W., Roe J.A., Feigon J. (1993). Thrombin-binding DNA aptamer forms a unimolecular quadruplex structure in solution. Proc. Natl. Acad. Sci. USA.

[20] Demkovičová E., Bauer Ľ., Krafčíková P., Tlučková K., Tóthova P., Halaganová A., Valušová E., Víglaský V. (2017). Telomeric G-Quadruplexes: From Human to Tetrahymena Repeats. J. Nucleic Acids.

[21] Greider C.W., Blackburn E.H. (1989). A telomeric sequence in the RNA of Tetrahymena telomerase required for telomere repeat synthesis. Nature.

[22] Wang Y., Patel D.J. (1994). Solution structure of the Tetrahymena telomeric repeat d(T2G4)4 G-tetraplex. Structure.

[23] Ambrus A., Chen D., Dai J., Bialis T., Jones R.A., Yang D. (2006). Human telomeric sequence forms a hybrid-type intramolecular G-quadruplex structure with mixed parallel/antiparallel strands in potassium solution. Nucleic Acids Res..

[24] Mathad R.I., Hatzakis E., Dai J., Yang D. (2011). c-MYC promoter G-quadruplex formed at the 5′-end of NHE III1 element: Insights into biological relevance and parallel-stranded G-quadruplex stability. Nucleic Acids Res..

[25] Brcic J., Plavec J. (2018). NMR structure of a G-quadruplex formed by four d(G4C2) repeats: Insights into structural polymorphism. Nucleic Acids Res..

[26] Tlučková K., Marušič M., Tóthová P., Bauer L., Šket P., Plavec J., Viglasky V. (2013). Human papillomavirus G-quadruplexes. Biochemistry.

[27] Kocman V., Plavec J. (2014). A tetrahelical DNA fold adopted by tandem repeats of alternating GGG and GCG tracts. Nat. Commun..

[28] Trachman R.J., Autour A., Jeng S.C.Y., Abdolahzadeh A., Andreoni A., Cojocaru R., Garipov R., Dolgosheina E.V., Knutson J.R., Ryckelynck M. (2019). Structure and functional reselection of the Mango-III fluorogenic RNA aptamer. Nat. Chem. Biol..

[29] Sjekloća L., Ferré-D’Amaré A.R. (2019). Binding between G Quadruplexes at the Homodimer Interface of the Corn RNA Aptamer Strongly Activates Thioflavin T Fluorescence. Cell Chem. Biol..

[30] Anastassiou D. (2000). Frequency-domain analysis of biomolecular sequences. Bioinformatics.

